# Library of the Medical College of Ohio

**Published:** 1834

**Authors:** 


### III.—Library of the Medical College of Ohio.

The library of the college embraces upwards of 1200 volumes, chiefly European. They have been purchased, exclusively, with moneys given by the state; and are designed for the promotion of medical science. The university of Pennsylvania has no medical library, but the Pennsylvania hospital, connected in various ways with the medical department of the University, possesses an extensive and valuable collection. The Transylvania school in Kentucky, has a library of more than 3000 volumes.

Both the managers of the hospital in Philadelphia, and the professors of the college in Lexington, have established a library ticket, which they sell by the year, or at shorter periods, to students of medicine and *physicians*, and thus lay open to the profession, in those cities, and the surrounding country, the rich stoies of medical and other scientific knowledge which are there accumulated; at the same time, causing the books, before they become obsolete, as all medical writings (including even our own) sooner or later must, to bring in something for the purchase of the new works, by which they are ultimately to be superseded.

But what has been and still is, the policy of the trustees and professors of the Medical College of Ohio, in reference to its library, purchased as we have said with the moneys of the state? It is to lock it up a prey for worms and spiders. During the session of four months, when students of medicine have no time to read any but the common text books, they have access to the collection, but for the other eight months of the year, not one of them can obtain a single volume, and no physician of Ohio, can on any terms procure admission within the walls of the library, either in the sessions or vacations of the college. Thus many students from a distance, who might, in the latter periods of their pupilage, be induced to remain in Cincinnati, and read such books as cannot be found in the libraries of their preceptors, are kept from doing so; and many young or inquisitive physicians of Ohio, who might, at a small expense, prosecute their studies, beyond the narrow limits, where the majority stop, are precluded from these advantages, while an annual source of revenue to the institution is entirely lost.

It is difficult to comprehend the reasons for this policy. It does not arise from apprehended danger to the books, if they were given out to resident summer pupils, and to the physicians of the city and state, under suitable pledges, for in the winter, students of every kind, from any distance, who are in attendance on the lectures, obtain books, by simply depositing their promissory notes, and paying less than would be willingly paid by students in vacation and by physicians; nor is it persevered in, from its not having been presented to the consideration of the Faculty and Trustees, as this has been done. In conclusion, we shall add the following fact. A physician long resident in the city, and desirous of access to the library, in the spring of 1833, made a special application to the Board of Trustees. It was granted, but *limited to the vacation of that year*, and although a property holder, he could not get out a single volume, till he executed a bond, with security, in a penalty of two hundred dollars! Any physician of the state of Kentucky can obtain books from the Medical Library of Transylvania University, for the whole year, by paying eight dollars for a ticket; and if he reside in Lexington, may spend a part of every day, in reference, and research. It is not surprising, that the physicians of that state are attached to its institution, and that the number of its pupils is constantly more than double that of the Medical College of Ohio.

